# Perceived Stress, Resilience, and Anxiety Among Pregnant Chinese Women During the COVID-19 Pandemic: Latent Profile Analysis and Mediation Analysis

**DOI:** 10.3389/fpsyg.2021.696132

**Published:** 2021-07-22

**Authors:** Zheng Luo, Yaqing Shen, Jiajia Yuan, Yifan Zhao, Zhengkui Liu, Fangfang Shangguan

**Affiliations:** ^1^Beijing Key Laboratory of Learning and Cognition, School of Psychology, Capital Normal University, Beijing, China; ^2^CAS Key Laboratory of Mental Health, Institute of Psychology, Chinese Academy of Sciences, Beijing, China; ^3^Department of Psychology, University of Chinese Academy of Sciences, Beijing, China

**Keywords:** perceived stress, person-centered, resilience, anxiety, pregnant women, COVID-19

## Abstract

**Objective:** The coronavirus disease 2019 (COVID-19) pandemic has posed a major threat to pregnant women's mental health. This study aimed to characterize the patterns of perceived stress in pregnant Chinese women during the COVID-19 pandemic, to examine the profile differences on anxiety and resilience, and to investigate whether the differences in these profiles on anxiety were mediated by resilience.

**Methods:** From February 28, 2020 to April 26, 2020, a sample of 2,116 pregnant Chinese women who participated in online crisis interventions completed an online self-reporting questionnaire assessing their demographic characteristics, perceived stress, resilience, and anxiety.

**Results:** Latent profile analysis (LPA) on two stress dimensions [perceived helplessness (HEL) and perceived self-efficacy (SEL)] indicated four perceived stress profiles: adaptive (33.7% of the sample), resistant (44.6%), insensitive (19.1%), and sensitive (2.6%). The women with both adaptive and insensitive profiles had the lowest levels of anxiety, whereas those with the resistant profile had the lowest levels of resilience. Multicategorical mediation analysis showed that resilience partially mediated the differences in the pregnant women's anxiety between the adaptive/insensitive and resistant profile.

**Conclusion:** This study showed the heterogeneity in the perceived stress patterns of pregnant women during the COVID-19 pandemic, revealing the internal mechanisms of pregnant women's anxiety using a person-centered approach, and provided initial evidence guiding the development of differentiated stress interventions to alleviate pregnant women's anxiety during the pandemic.

## Introduction

Since late December 2019, a novel coronavirus disease 2019 (COVID-19) has spread rapidly in China and between countries, with high morbidity and mortality rates. It was declared as a global pandemic by the WHO on March 11, 2020. Emerging evidence from around the world suggests that pregnant women during the COVID-19 pandemic are experiencing elevated anxiety (Berthelot et al., 2020; Preis et al., 2020a; Wu et al., 2020), a well-documented risk factor during pregnancy for adverse obstetric and neonatal outcomes (Stein et al., 2014; Vollrath et al., 2016; Hasanjanzadeh and Faramarzi, 2017). During the initial phase of the COVID-19 outbreak in China, pregnant women reported higher levels of anxiety after the announcement of human-to-human transmission (Wu et al., 2020). A meta-analysis showed that the prevalence rate of anxiety among pregnant women during the COVID-19 pandemic was 37% (95% CI: 25–49%) (Yan et al., 2020). To date, approximately 20% of pregnant women in China have suffered from anxiety (Ding et al., 2021; Jiang et al., 2021) and 43.3% of pregnant women in the USA experience moderate-to-severe anxiety (Preis et al., 2020a). Additionally, in Iran, approximately 21% of pregnant women reported pregnancy-related anxiety (Hamzehgardeshi et al., 2021) and the same percentage of women in the third trimester of pregnancy had severe health anxiety (Saadati et al., 2021). Anxiety among pregnant women during the global pandemic should be one of the priorities of public health.

Anxiety among pregnant women may be affected by the COVID-19 pandemic due to their perceived stress (Preis et al., 2020a; Romero-Gonzalez et al., 2021). Perceived stress is experiencing distress while responding to stressors. Cumulative evidence (e.g., Hewitt et al., 1992; Martin et al., 1995; Leung et al., 2010; Taylor, 2015) has indicated that perceived stress is a multidimensional construct containing two dimensions: perceived helplessness (HEL; also known as “negative feelings” and “perceived distress”) and perceived self-efficacy (SEL; also known as “positive feelings” and “perceived coping ability”). The former refers to negative affective reactions and general distress, whereas the latter refers to the perception of an ability to cope with existing stressors. In terms of COVID-19, the perceived stress of pregnant women highlights the extent to which they believe they can control unexpected or difficult events or emotions resulting from the pandemic, such as quarantine and social distancing precautions, the uncertainty of viral infection, the lack of social support (Ding et al., 2021; Hamzehgardeshi et al., 2021), and their preparedness stress and perinatal infection stress (Preis et al., 2020b).

Research on the relation between perceived stress and anxiety among pregnant women during the COVID-19 pandemic overwhelmingly took a variable-centered approach that overlooked individual differences (e.g., Preis et al., 2020a; Romero-Gonzalez et al., 2021). Although some studies have found that both HEL and SEL can predict psychological problems (e.g., depression; Hewitt et al., 1992), others have found that HEL but not SEL is related to psychological problems (Martin et al., 1995). One reason for this inconsistent pattern is the heterogeneous distribution of the study samples. “Adaptability and resistance to stress are fundamental prerequisites for life” (Selye, 1950). Pregnant women may have adaptive or resistant responses to stressors during the COVID-19 pandemic. Meanwhile, because of individual differences in stress sensitivity (Zubin and Spring, 1977), some pregnant women may be stress sensitive (i.e., responding to stress with heightened levels of negative emotions) and some others may be stress insensitive. Accordingly, the perceived stress of pregnant women associated with the pandemic may be clustered according to different dimensions (i.e., HEL and SEL). To explore the patterns or profiles of the combination of HEL and SEL among pregnant women during the COVID-19 pandemic and how the patterns predict anxiety among pregnant women, a person-centered analysis approach was adopted in the current study.

As a person-centered analysis technique, the latent profile analysis (LPA) groups individuals into latent classes or profiles or subgroups according to the correlations on continuous variables. The LPA results reveal a typical co-occurrence of HEL and SEL among subgroups, which makes it possible to identify the patterns of pregnant women's perceived stress during the COVID-19 pandemic. This understanding can then be used to recognize the group to which each pregnant woman belongs and in turn to guide appropriate intervention efforts aimed at each group's unique needs rather than the target variables.

A few studies have explored the profiles of perceived stress (e.g., Berlin et al., 2012; Liao et al., 2018; Langford et al., 2019) using LPA. Most of these studies focused on how individuals evaluated different stressors rather than using a global stress appraisal. “Low stress” (Berlin et al., 2012) or “ordinary” (Liao et al., 2018) or “normative” (Langford et al., 2019) profiles characterized by relatively low levels of perceived stress indicators have been consistently identified from a prior work. One study conducted by Fernández et al. (2020) identified three latent classes of psychological distress associated with COVID-19 quarantine among Argentine volunteers. The majority of the individuals could be classified into mild (40.9%) and severe classes (41.0%). To our knowledge, no study has examined the perceived stress profile of pregnant women using LPA. Meanwhile, although the evidence has shown that there are differences in depression levels among different perceived stress profiles (Liao et al., 2018), it is still not clear whether there are differences in anxiety among different perceived stress profiles of pregnant women exposed to the COVID-19 epidemic.

Resilience is a “dynamic process encompassing positive adaptation within the context of significant adversity” (Luthar et al., 2000). Many personal abilities and traits, such as optimism (Connor and Davidson, 2003) and tenacity (Rutter, 1985), are associated with resilience. There is no comprehensive and unifying theoretical framework in the field of resilience research, and the causal trajectory is controversial (Fletcher and Sarkar, 2013). Some cross-sectional studies have investigated the mediating role (questions of “how”; e.g., Tam et al., 2020), moderating role (questions of “when”; e.g., Tsourtos et al., 2019), or both roles (Anyan and Hjemdal, 2016; Ma et al., 2019) of resilience in the relation between stress and psychiatric symptoms. The mediating role of resilience between stress and anxiety symptoms has been proven, but the moderating role of resilience is uncertain. For example, Ma et al. (2019) found that resilience was both a mediator and a modifier of the association between stress and prenatal anxiety. Anyan and Hjemdal (2016) found that resilience partially mediated the relation between stress and symptoms of anxiety. However, it did not moderate the influence of stress on symptoms of anxiety.

The compensatory model of resilience (Zimmerman et al., 1998; Zimmerman and Brenner, 2010) holds that the direct effects of resilience counterbalance the direct negative effects of risk factors such as stress, which suggests a mediating role of resilience between stress and anxiety. Empirical studies have indicated that individuals respond to different life experiences with varying degrees of resilience (Waller, 2001). Stress can have an adverse impact on an individual's resilience (Bonanno and Mancini, 2008), and more experience with adversities is associated with lower resilience among pregnant and postpartum women (Harville et al., 2010). Meanwhile, a meta-analysis revealed that resilience is negatively associated with psychiatric symptoms (e.g., anxiety; Hu et al., 2015). Based on the compensatory model of resilience and the empirical evidence linking stress, resilience, and anxiety symptoms, this study concentrates on the mediating role of resilience between the perceived stress and symptoms of anxiety (i.e., how does the perceived stress result in symptoms of anxiety *via* resilience?) among pregnant women during the COVID-19 pandemic. Pregnant women with high resilience showed lower levels of psychological distress during the COVID-19 pandemic (Chasson et al., 2020). However, whether group differences in perceived stress profiles on pregnant women's anxiety during the COVID-19 pandemic were mediated by resilience has not yet been specifically investigated.

The present study aimed to identify integrative stress profiles consisting of two perceived stress dimensions and to explore the relationship among stress profiles, resilience, and anxiety of pregnant Chinese women using LPA during the COVID-19 pandemic. We hypothesized that (1) there may be perceived stress profiles reflecting different combinations of HEL and SEL. We employed an exploratory approach and therefore made no hypothesis about the number of perceived stress profiles. (2) There were significant differences in anxiety and resilience among the different stress profiles. Profiles with lower HEL have lower levels of anxiety and higher levels of resilience. (3) Resilience would mediate the effect of stress profile differences on anxiety. That is, the differences in anxiety between a profile with lower HEL and other profiles could be explained by the former group's higher resilience.

## Methods

### Participants and Procedures

This study is part of a WeChat psychological crisis intervention program initiated by the Institute of Psychology, Chinese Academy of Sciences, that aimed to help pregnant Chinese women cope with stress during the COVID-19 pandemic. Pregnant women who attended regular examinations at medical institutions in Wuhan, Beijing, Lanzhou, and other cities of China were recruited to scan the QR code generated by an online survey platform to complete the survey. The inclusion criteria were at all stages of pregnancy, more than 18 years old, being able to read and write in Chinese and no infection with COVID-19. Pregnant women with a history of mental illness were excluded from the study (*n* = 7). Pregnant women participated voluntarily in this study and provided an informed consent between February 28, 2020 and April 26, 2020. Ethics approval for the study was obtained from the Institutional Review Board of Institute of Psychology, Chinese Academy of Sciences.

### Measurements

#### Perceived Stress

A 10-item Perceived Stress Scale (PSS-10) was used to assess persons' perceptions of situations in their life in terms of uncontrollability, unpredictability, and overload (Cohen et al., 1983; Cohen and Williamson, 1988). It was divided into two dimensions: HEL (items 1, 2, 3, 6, 9, and 10) and SEL (items 4, 5, 7, and 8, reverse scoring) (Leung et al., 2010; Taylor, 2015). The items were rated on a five-point Likert scale from 0 (“never”) to 4 (“very often”). Higher scores on these two dimensions indicated a higher negative emotion perception and a stronger sense of an inability to cope with stress. This scale has been validated among pregnant women (Monique et al., 2010). In this study, the Cronbach's α was 0.85.

#### Resilience

A 10-item Connor-Davidson resilience scale (CD-RISC) was applied to assess the adaptability to stress (Connor and Davidson, 2003; Campbell-Sills and Stein, 2007). The 10-item CD-RISC is a unidimensional scale rated on a five-point Likert scale ranging from 0 (“not true at all”) to 4 (“true nearly all of the time”). The 10-item CD-RISC has been validated among pregnant women (Levey et al., 2019). In this study, the Cronbach's α was 0.96.

#### Anxiety

A seven-item Generalized Anxiety Disorder scale (GAD-7) was used to measure the severity of anxiety symptoms, with a four-point Likert scale response ranging from 0 (“almost never”) to 3 (“almost always”). GAD-7 was initially developed for screening the generalized anxiety disorder (GAD) and assessing the severity of symptoms in a primary care patient sample (Spitzer et al., 2006). It has also been validated or used for assessing anxiety symptoms in the general population (Löwe et al., 2008; Solomou and Constantinidou, 2020), patients with cancer (Lundt and Jentschke, 2019), and pregnant women (e.g., Barthel et al., 2014; Rosenthal et al., 2015). Internal consistency was obtained as satisfactory in this study (Cronbach's α = 0.92).

### Statistical Analysis

SPSS 25.0 and Mplus 7.0 were used in the analyses. First, descriptive statistics and Pearson correlation analysis for all of the variables were applied. Second, LPA was utilized to identify latent stress profiles according to HEL and SEL. The one- to six-class groups were applied and compared based on a set of fit statistics. A good model fit is indicated by (1) lower comparative values of the Akaike information criteria (AIC), the Bayesian information (BIC), and the adjusted BIC (ABIC) values, as well as higher values of entropy with numbers closer to 1; (2) a significant Lo-Mendell-Rubin likelihood ratio test (LMR LR) and the Vuong-Lo-Mendell-Rubin test (VLMR). Third, after determining the best class solution, univariate ANOVAs and *post-hoc* tests were applied to compare the differences among the stress profiles with respect to the two stress dimensions and resilience and anxiety.

Following Hayes and Preacher (2014), a multicategorical mediating model was constructed through structural equation modeling (SEM) to investigate whether the differences among the perceived stress profiles (multicategorical variables) on anxiety (latent variable, the measured indicators were seven items of GAD-7) could be explained by resilience (latent variable, the measured indicators were five parcels of items of CD-RISC). The criteria for good model fit indices for SEM were as follows: χ^2^/df ≤ 5.000, comparative fit index (CFI), Tucker–Lewis index (TLI) ≥ 0.900, standardized root mean square residual (SRMR) ≤ 0.080, and root mean square error of approximation (RMSEA) ≤ 0.080 (Kline, 2011; Hoyle, 2012).

## Results

### Sample Description

The final participants included 2,116 pregnant women whose average age was 30.24 years old (SD = 3.97, range = 19–47 years). Among the participants, 22.7% were in the first trimester, 23.8% in the second trimester, and 53.5% in the third trimester. The majority of participants were married (98.2%) and of Han nationality (95.8%). In terms of geography, 38.5% were from Beijing, 32.7% were from Hubei (among them, 99.1% were from Wuhan), 25.6% were from Gansu, 2.4% were from Hebei, and 0.8% were from the other provinces in China. Regarding their education levels, 11% had completed graduate studies or above, 44.1% had completed university, 28.7% had completed junior college, and 16.2% had completed senior high school or less. In terms of economic status, 12.6% of the participants' annual family income exceeded 300,000 RMB, and 31.24% of the participants' annual family income was <80,000 RMB. A total of 17.63% of the sample reported to have pregnancy complications.

### Descriptive Statistics

Means, SDs, and correlations for all of the variables are presented in Table 1. The results showed that anxiety was positively associated with HEL (*p* < 0.001) but not related to SEL (*p* > 0.05). Resilience was negatively associated with HEL, SEL, and anxiety (*p* < 0.001).

**Table 1:** Descriptive statistics and correlation matrix of all variables.

	***M***	***SD***	**1**	**2**	**3**
1 HEL	1.104	0.820	–		
2 SEL	1.752	1.131	−0.274 ^***^	–	
3 Resilience	2.989	0.790	−0.299 ^***^	−0.266 ^***^	–
4 Anxiety	0.350	0.472	0.581 ^***^	0.010	−0.371 ^***^

*HEL, perceived helplessness; SEL, perceived self-efficacy*.

*** *p < 0.001*.

### Perceived Stress Profiles

The fit indices of the six LPA models are reported in Table 2. The four-profile model had lower AIC, BIC, and ABIC values than the three-profile model and had significant values of *p* for LMR LR and VLMR. The five-profile model had significant values of *p* for LMR LR and VLMR, and lower AIC, BIC, and ABIC values than the four-profile model, but the downtrend of AIC, BIC, and ABIC became slow, and the entropy was less than that of the four-profile model. In addition, considering the simplicity and relative distinguishability of the model, we chose the four-profile solution as the final model.

**Table 2:** Model fit indices for one to six profile solutions of perceived stress.

**Model**	**AIC**	**BIC**	**ABIC**	**Entropy**	**LMR LR (*p*)**	**VLMR (*p*)**
1-profile	12015.895	12038.524	12025.816			
2-profile	11232.247	11271.848	11249.608	0.899	0.000	0.000
3-profile	10945.377	11001.950	10970.179	0.720	0.000	0.000
**4-profile**	**10622.749**	**10696.293**	**10654.991**	**0.824**	**0.000**	**0.000**
5-profile	10550.852	10641.369	10590.535	0.815	0.029	0.033
6-profile	10430.187	10537.675	10477.310	0.809	0.050	0.055

*AIC, Akaike information criterion; BIC, Bayesian information criterion; ABIC, Adjusted BIC; LMR LR, Lo-Mendell-Rubin likelihood ratio test; VLMR, Vuong-Lo-Mendell-Rubin test. Indices of the best-fitting model are in boldface*.

**Figure 1:**
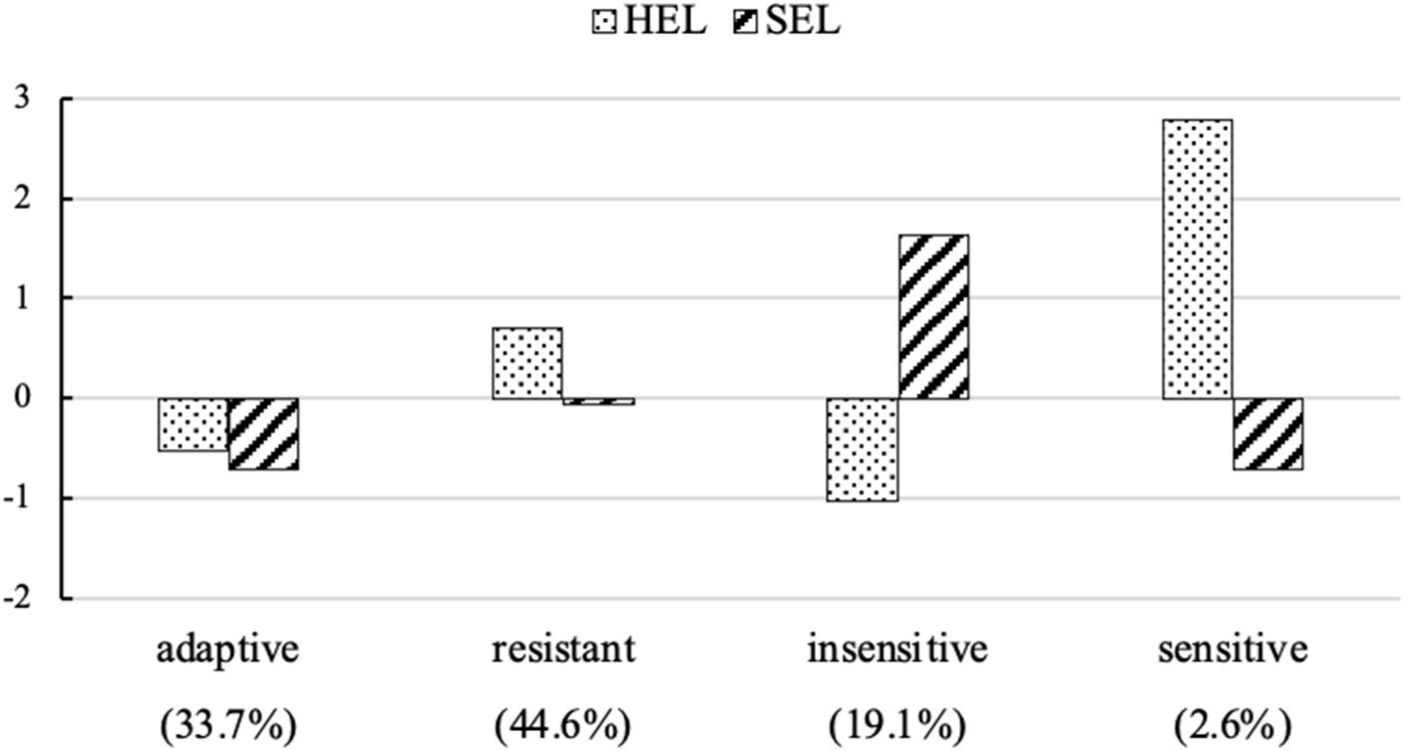
The four perceived stress profiles and relative size of the profiles. HEL, perceived helplessness; SEL, perceived self-efficacy. Profile indicator variables were standardized values.

Figure 1 and Table 3 summarize the characteristics of the four stress profiles identified using standardized scores. The profiles differed from one another with respect to the two perceived stress dimensions, characterized by low HEL/low SEL, high HEL/moderate SEL, low HEL/high SEL, and very high HEL/low SEL. We labeled them as adaptive (33.7%), resistant (44.6%), insensitive (19.1%), and sensitive (2.6%).

**Table 3:** The dimensions of perceived stress, resilience, and anxiety for four perceived stress profiles.

	**Adaptive**	**Resistant**	**Insensitive**	**Sensitive**	**F (*p*)**	**Effect size**
HEL	−0.608_c_	0.732_b_	1.015_d_	2.808_a_	2301.299 ^***^	0.766
SEL	0.764_c_	0.076_b_	1.629_a_	0.758_c_	1821.654 ^***^	0.721
Resilience	3.371_a_	2.721_c_	2.946_b_	2.942_b_	106.232 ^***^	0.131
Anxiety	0.142_c_	0.565_b_	0.143_c_	0.886_a_	208.621 ^***^	0.229

*The dimensions of perceived stress are presented as standardized z-scores. Values with different subscripts in the same row represent significantly different values based on Tukey's honest significant difference (HSD) tests for HEL, SEL, resilience, and anxiety. HEL, perceived helplessness; SEL, perceived self-efficacy*.

*** *p < 0.001*.

### Profile Differences in Resilience and Anxiety

The differences in resilience and anxiety among the four profiles were examined by using ANOVAs. The adaptive profile had the highest resilience. The insensitive and sensitive profile had middle-level resilience. The resistant profile had the lowest resilience. The profiles also differed overall in terms of anxiety. The sensitive profile had the highest anxiety. The resistant profile had middle-level anxiety. The adaptive and insensitive profiles had the least amount of anxiety (Table 3).

### Mediated Effects of Resilience

Three dummy variables (D1, D2, and D3) were created by using indicator coding to represent the four stress profiles. The resistant profile served as a reference group and was assigned a score of “0” across all variables. The adaptive, insensitive, and sensitive profiles were coded as “100,” “010,” and “001,” respectively. Adding the pregnant women's age, gestational age, number of births, and physical disease as covariates, these dummy variables were then entered into the SEM to test whether the differences in anxiety between the adaptive/insensitive/sensitive and resistant profiles were due to the differences in resilience and resilience's subsequent effect on anxiety. Compared with the resistant profile, the relative direct and indirect effects for the other profiles were calculated. The significance of each relative indirect path was tested by using the bootstrapping method (1,000 samples). The mediation model was fitted ideally with χ^2^/df = 4.16, CFI = 0.985, TLI = 0.980, RMSEA = 0.039 [0.035, 0.042], SRMR = 0.019.

**Figure 2:**
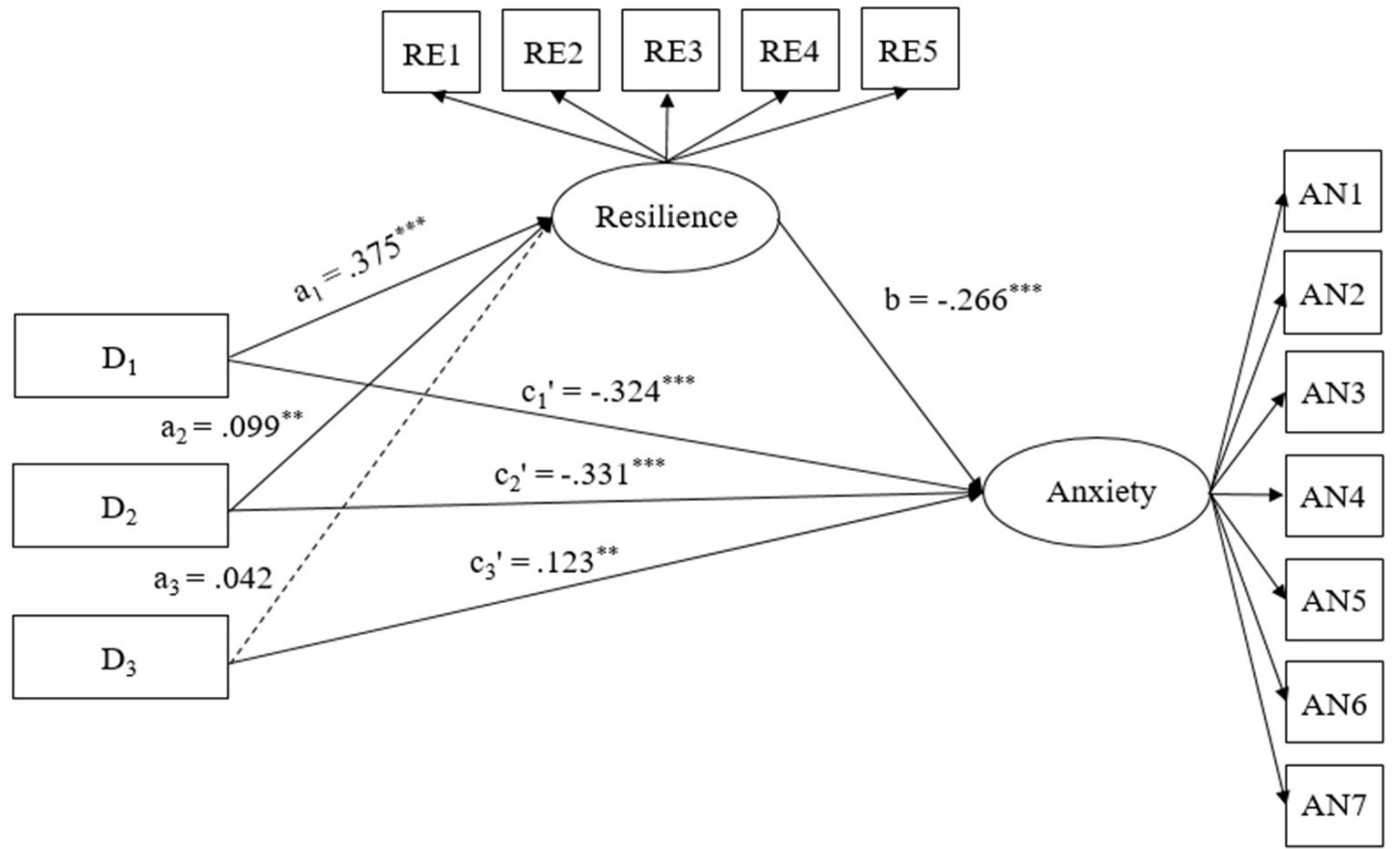
Model of the mediation role of resilience in association between perceived stress profiles and anxiety. D_1_, D_2_, and D_3_ were dummy variables to represent the perceived stress profiles. The resistant profile served as the reference group and were assigned a score of “0” across the three variables. The adaptive, insensitive, and sensitive profiles were assigned with respective scores of “100,” “010,” and “001” across D_1_, D_2_, and D_3_. RE1-RE5 = five parcels of resilience; AN1-AN7 = seven items of anxiety. ***p < 0.001.

According to Hayes and Preacher (2014), a1, a2, and a3 and c1', c2', and c3' correspond to the differences in the adaptive, insensitive, and sensitive profiles for resilience and anxiety, respectively, relative to the resistant profile. b quantifies the effect of resilience on anxiety (Figure 2). The bootstrap CI indicated a significant relative indirect effect of D1 and D2 on anxiety *via* resilience (for D1, β = −0.10, *E* = 0.01, 99% CI [−0.13, −0.08]; for D2, β = −0.03, *E* = 0.01, 99% CI [−0.05, −0.01]), while the relative direct effect of D1 and D2 on anxiety was significant (for D1, β = −0.32, *E* = 0.02, 99% CI [−0.38, −0.28]; for D2, β = −0.33, *E* = 0.02, 99% CI [−0.38, −0.28]). The results suggested that compared with the resistant profile, the adaptive profile and insensitive profile led to significantly lower levels of anxiety *via* higher levels of resilience. The examination of the proportion of relative mediation effects showed that 23.6% (adaptive profile) and 7.3% (insensitive profile) of the relative total effect on anxiety were mediated by resilience. Meanwhile, the CIs spanned zero, indicating that the relative indirect effects of D3 (the sensitive profile, relative to the resistant profile) on anxiety *via* resilience were not significant. The relative direct effect of D3 on anxiety was significant (β = 0.12, *E* = 0.04, 99% CI [0.03, 0.23]).

## Discussion

We found that the perceived stress among pregnant women during the COVID-19 pandemic could be classified into four profiles: adaptive (low HEL/low SEL), resistant (high HEL/moderate SEL), insensitive (low HEL/high SEL), and sensitive (very high HEL/low SEL), which differed from one another in terms of anxiety and resilience. The differences in the pregnant women's anxiety between the adaptive/insensitive and resistant stress profiles could be explained by the former groups' higher resilience.

The resistant stress profile occurs most frequently among pregnant women. This finding is partly in accordance with some previous research findings. For example, Lee et al. (2006) reported that pregnant women in Hong Kong, China, tended to display obvious stress responses during the 2003 SARS outbreak period, such as overestimation of the risk of infection. Meanwhile, they showed a coping ability by adopting behavioral strategies to mitigate their risk. The adaptive stress profile is similar to the “low stress” profile described by Berlin et al. (2012) and the “ordinary” profile described by Liao et al. (2018). Pregnant women in this group felt less distress and had a better sense of coping even during the COVID-19 pandemic. The opposite features existed in the insensitive and sensitive profiles. However, the number of pregnant women in both groups was relatively small.

In general, the pregnant women with a sensitive profile had the highest levels of anxiety, followed by the pregnant women with a resistant profile, an insensitive profile, and an adaptive profile. This suggested that a higher HEL is associated with an increased risk of anxiety. This finding is partly in line with a previous study; that is, only HEL is related to psychiatric symptoms between HEL and SEL for women (Martin et al., 1995) and for men (Hewitt et al., 1992). Anxiety symptoms are characterized precisely by excessive negative emotion according to the *Diagnostic and Statistical Manual of Mental Disorders* (5th ed.; DSM-5; American Psychiatric Association, 2013). Therefore, pregnant women may respond with anxiety symptoms to their perception of distress. As expected, there was no association between SEL and anxiety in this study. This is consistent with a previous study showing that efficacy expectations (a perceived ability to cope with the situation) did not significantly predict anxiety (Dowd et al., 1985). One explanation for this finding might be that the relation between coping and anxiety is conditional. It would be affected by some moderators (Li and Miller, 2017), which requires additional research to achieve a better understanding.

An important finding of this research was that the effects of the differences between adaptive/insensitive and resistant profiles on anxiety were partially mediated by resilience. Compared with the resistant stress profile, the adaptive and insensitive stress profile led to significantly lower levels of anxiety *via* higher levels of resilience, which is also partly consistent with past dimension-approach research results (Anyan and Hjemdal, 2016; Ma et al., 2019) and supported by the compensatory model of resilience (Zimmerman et al., 1998). Compared with pregnant women in the resistant profile, pregnant women in the adaptive profile can better adapt to changes in the environment and their social life and have perceptions of fewer negative affective reactions and a high coping ability and self-efficacy. These factors may give pregnant women the resources to cope with stressors under many situations and develop resilience (Galatzer-Levy et al., 2013; Sagone and Caroli, 2013; Schwarzer and Warner, 2013). Pregnant women with an insensitive profile had lower perceptions of distress and coping ability, which may protect them from consuming too many available resources to deal with negative emotions, conducive to maintaining resiliency (Galatzer-Levy et al., 2013). Meanwhile, based on the compensatory model of resilience (Zimmerman et al., 1998) and empirical findings (e.g., Hjemdal et al., 2011), resilience can directly decrease the risk of anxiety. Therefore, compared to the resistant profile, the higher resilience of the adaptive profile and insensitive profile directly predicted lower levels of anxiety.

The study found that although women with a sensitive profile had a higher level of anxiety than women with a resistant profile, resilience did not mediate the effect of the difference on anxiety between the sensitive stress profile and the resistant stress profile. One possible reason may be that the sensitive profile had a very high HEL, which can directly lead to anxiety and need not be mediated by resilience. A possible effect of heightened stress sensitivity on affective disorder onset and susceptibility has been supported by previous studies (Bale, 2006). Another possible explanation is that there may be other mediators that explain the differences in anxiety between the sensitive and resistant profiles that were not considered in this study. However, this explanation is speculative. Additional studies are needed to verify the current findings.

To our knowledge, this is the first study to apply LPA to identify the patterns of pregnant women's perceived stress during the COVID-19 pandemic and to examine the effect of resilience on the association between the perceived stress profiles and anxiety from a person-centered approach. However, this study has some limitations that need to be addressed. First, this was a cross-sectional study, and there was an absence of a pre-pandemic control group, which prevented us from reaching any causal conclusions about the association of perceived stress with anxiety. Future research should conduct longitudinal studies to identify causal relationships. Second, a self-reported data collection method might affect the validity of the data. Future research should combine multiple methods, such as brain imaging and molecular biological techniques, to collect data. Meanwhile, qualitative interviews or focused group discussions could have added more value to the study by exploring the causes of pregnant women's anxiety and how they cope with it. Third, although our study was based on two important dimensions of perceived stress, it might not fully encompass the stress that the pregnant women experienced. Fourth, GAD-7 was originally designed for screening for GAD and assessing the severity of symptoms in a clinical sample (Spitzer et al., 2006) although it has also been confirmed or used to assess anxiety symptoms among pregnant women (e.g., Barthel et al., 2014; Rosenthal et al., 2015). Comparisons with previous studies should be made with caution because different studies may assess different aspects of anxiety and its severity.

Despite the limitations, our findings might have important implications for medical staff to develop more effective crisis intervention programs to alleviate pregnant women's anxiety during a period of crisis. Pregnant women with different HEL/SEL patterns might have different levels of anxiety, which suggests differentiated clinical psychological nursing and interventions to balance the needs of all pregnant women. We encourage future anxiety interventions of pregnant women aimed at managing stress from a specific person-centered mode. Pregnant women in the adaptive group have low levels of HEL and SEL, which can help them deal with crises. For these pregnant women, additional psychological interventions are not needed. For pregnant women in the insensitive group, the main goal is to increase their perceptions of coping ability, i.e., general self-efficacy, through health education emphasizing high performance accomplishments, vicarious experiences, verbal persuasion, etc. (Bandura, 1977). Pregnant women in the resistant group and sensitive group should be the focus of crisis intervention. The primary goal is to relieve their high HEL, such as being instructed to use various positive emotion regulation strategies (e.g., Garnefski et al., 2002) and through cognitive interventions (e.g., Zemestani and Fazeli Nikoo, 2020). Meanwhile, pregnant women with adaptive and insensitive patterns could experience decreases in anxiety *via* resilience. Thus, our results offer a reasonable basis for further developing resilience-specific crisis interventions that would be more targeted and thus increase their effectiveness. For example, it could help pregnant women to develop meaningful connections with family or friends and perceive increased social support to improve their resilience and reduce their anxiety.

## Data Availability Statement

The raw data supporting the conclusions of this article will be made available by the authors, without undue reservation.

## Ethics Statement

The studies involving human participants were reviewed and approved by the Institutional Review Board of Institute of Psychology, Chinese Academy of Sciences. The patients/participants provided their written informed consent to participate in this study.

## Author Contributions

ZLu, FS, and ZLi: study design, critical revision of the manuscript, and approval of the final version for publication. ZLu, YS, JY, and YZ: analysis and interpretation of data. ZLu and YS: drafting of the manuscript. All authors contributed to the article and approved the submitted version.

## Conflict of Interest

The authors declare that the research was conducted in the absence of any commercial or financial relationships that could be construed as a potential conflict of interest.

## References

[B1] American Psychiatric Association (2013). Diagnostic and Statistical Manual of Mental Disorders. (5th ed.). Washington, DC: American Psychiatric Association. 10.1176/appi.books.9780890425596

[B2] Anyan F. Hjemdal O. (2016). Adolescent stress and symptoms of anxiety and depression: resilience explains and differentiates the relationships. J. Affective Disord. 203, 213–220. 10.1016/j.jad.2016.05.031 27310100

[B3] Bale T. L. (2006). Stress sensitivity and the development of affective disorders. Horm. Behav. 50, 529–533. 10.1016/j.yhbeh.2006.06.033 16901485

[B4] Bandura A. (1977). Self-efficacy: toward a unifying theory of behavioral change. Psychol. Rev. 84, 191–215. 10.1037/0033-295X.84.2.191 847061

[B5] Barthel D. Barkmann C. Ehrhardt S. Bindt C. (2014). Psychometric properties of the 7-item Generalized Anxiety Disorder scale in antepartum women from Ghana and Côte d'Ivoire. J. Affective Disord. 169, 203–211. 10.1016/j.jad.2014.08.004 25212996

[B6] Berlin K. S. Rabideau E. M. Hains A. A. (2012). Empirically derived patterns of perceived stress among youth with type 1 diabetes and relationships to metabolic control. J. Pediatr. Psychol. 37, 990–998. 10.1093/jpepsy/jss080 22753443

[B7] Berthelot N. Lemieux R. Garon-Bissonnette J. Drouin-Maziade C. Martel É. Maziade M. (2020). Uptrend in distress and psychiatric symptomatology in pregnant women during the COVID-19 pandemic. Acta Obstetricia et Gynecol. Scand. 99, 848–855. 10.1111/aogs.13925 32449178

[B8] Bonanno G. A. Mancini A. D. (2008). The human capacity to thrive in the face of potential trauma. Pediatrics 121, 369–375. dio:10.1542/peds.2007-1648 18245429 10.1542/peds.2007-1648

[B9] Campbell-Sills L. Stein M. B. (2007). Psychometric analysis and refinement of the connor–davidson resilience scale (CD-RISC): validation of a 10-item measure of resilience. J. Trauma. Stress. 20, 1019–1028. 10.1002/jts.20271 18157881

[B10] Chasson M. Taubman-Ben-Ari O. Abu-Sharkia S. (2020). Jewish and arab pregnant women's psychological distress during the covid-19 pandemic: the contribution of personal resources. Ethnic Health. 12, 1–13. 10.1080/13557858.2020.1815000 32877202

[B11] Cohen S. Kamarck T. Mermelstein R. (1983). A global measure of perceived stress. J. Health. Soc. Behav. 24, 385–396. 10.2307/2136404 6668417

[B12] Cohen S. Williamson G. M. (1988). Perceived stress in a probability sample of the United States, in The Social Psychology of Health: Claremont Symposium on Applied Social Psychology, eds Spacapan S. Oskamp S. (Newbury Park, CA: Sage), 31–67.

[B13] Connor K. M. Davidson J. R. T. (2003). Development of a new resilience scale: the Connor-Davidson Resilience Scale (CD-RISC). Depress. Anxiety. 18, 76–82. 10.1002/da.10113 12964174

[B14] Ding W. Lu J. Zhou Y. Wei W. Zhou Z. Chen M. (2021). Knowledge, attitudes, practices, and influencing factors of anxiety among pregnant women in Wuhan during the outbreak of COVID-19: a cross-sectional study. BMC Pregnancy Childb. 21:80. 10.1186/s12884-021-03561-7 33494723 PMC7829651

[B15] Dowd E. T. Claiborn C. D. Milne C. R. (1985). Anxiety, attributional style, and perceived coping ability. Cognitive Ther. Res. 9, 575–582. 10.1007/BF01173010

[B16] Fernández R. S. Crivelli L. Guimet N. M. Allegri R. F. Pedreira M. E. (2020). Psychological distress associated with covid-19 quarantine: latent profile analysis, outcome prediction and mediation analysis. J. Affective Disord. 277, 75–84. 10.1016/j.jad.2020.07.133 32799107 PMC7413121

[B17] Fletcher D. Sarkar M. (2013). Psychological resilience: a review and critique of definitions, concepts, and theory. Eur. Psychol. 18, 12–23. 10.1027/1016-9040/a000124

[B18] Galatzer-Levy I. R. Brown A. D. Henn-Haase C. Metzler T. J. Neylan T. C. Marmar C. R. (2013). Positive and negative emotion prospectively predict trajectories of resilience and distress among high-exposure police officers. Emotion. 13, 545–553. 10.1037/a0031314 23339621 PMC3974969

[B19] Garnefski N. Van Den Kommer T. Kraaij V. Teerds J. Legerstee J. Onstein E. (2002). The relationship between cognitive emotion regulation strategies and emotional problems: comparison between a clinical and a non-clinical sample. Eur. J. Personality. 16, 403–420. 10.1002/per.458

[B20] Hamzehgardeshi Z. Omidvar S. Amoli A. A. Firouzbakht M. (2021). Pregnancy-related anxiety and its associated factors during COVID-19 pandemic in Iranian pregnant women: a web-based cross-sectional study,BMC Pregnancy Childb. 21:208. 10.1186/s12884-021-03694-9 33722198 PMC7957463

[B21] Harville E. W. Xiong X. Buekens P. Pridjian G. Elkind-Hirsch K. (2010). Resilience after hurricane Katrina among pregnant and postpartum women. Women Health Iss. 20, 20–27. 10.1016/j.whi.2009.10.002 20123173 PMC2822707

[B22] Hasanjanzadeh P. Faramarzi M. (2017). Relationship between maternal general and specific-pregnancy stress, anxiety, and depression symptoms and pregnancy outcome. J. Clin. Diagn. Res. 11, VC04–VC07. 10.7860/JCDR/2017/24352.9616 28571243 PMC5449889

[B23] Hayes A. F. Preacher K. J. (2014). Statistical mediation analysis with a multicategorical independent variable. Brit. J. Math. Stat. Psy. 67, 451–470. 10.1111/bmsp.12028 24188158

[B24] Hewitt P. L. Flett G. L. Mosher S. W. (1992). the perceived stress scale: factor structure and relation to depression symptoms in a psychiatric sample. J. Psychopathol. Behav. 14, 247–257. 10.1007/BF00962631

[B25] Hjemdal O. Vogel P. A. Solem S. Hagen K. Stiles T. C. (2011). The relationship between resilience and levels of anxiety, depression, and obsessive-compulsive symptoms in adolescents. Clin. Psychol. Psychot. 18, 314–321. 10.1002/cpp.719 20806419

[B26] Hoyle R. H. (2012). Model specification in structural equation modeling, in Handbook of Structural Equation Modeling, eds Hoyle R. H. Hoyle R. H. (New York, NY: Guilford Press), 126–144.

[B27] Hu T. Zhang D. Wang J. (2015). A meta-analysis of the trait resilience and mental health. Pers Individ Dif. 76:18–27. 10.1016/j.paid.2014.11.039

[B28] Jiang H. Jin L. Qian X. Xiong X. La X. Chen W.. (2021). Maternal mental health status and approaches for accessing antenatal care information during the COVID-19 epidemic in China: cross-sectional study. J. Med. Internet Res. 23:e18722. 10.2196/18722 33347423 PMC7817253

[B29] Kline R. B. (2011). Principles and Practice of Structural Equation Modeling (3rd ed.). New York, NY: Guilford Press.

[B30] Langford D. J. Cooper B. Paul S. Humphreys J. Hammer M. J. Levine J.. (2019). Distinct stress profiles among oncology patients undergoing chemotherapy. J. Pain Symptom Manage. 59, 646–657. 10.1016/j.jpainsymman.2019.10.025 31711968

[B31] Lee D. T. S. Sahota D. Leung T. N. Yip A. S. K. Lee F. F. Y. Chung T. K. H. (2006). Psychological responses of pregnant women to an infectious outbreak: a case-control study of the 2003 SARS outbreak in Hong Kong. J. Psychosom. Res. 61, 707–713. 10.1016/j.jpsychores.2006.08.005 17084150 PMC7094779

[B32] Leung D. Y. P. Lam T. Chan S. S. C. (2010). Three versions of Perceived Stress Scale: validation in a sample of Chinese cardiac patients who smoke. BMC Public Health 10, 513–519. 10.1186/1471-2458-10-513 20735860 PMC2939644

[B33] Levey E. J. Rondon M. B. Sanchez S. Williams M. A. Gelaye B. (2019). Psychometric properties of the Spanish version of the 10-item Connor Davidson Resilience Scale (CD-RISC) among adolescent mothers in Peru. J. Child Adolescent Trauma 14, 29–40. 10.1007/s40653-019-00295-9 33708280 PMC7900368

[B34] Li W. W. Miller D. J. (2017). The impact of coping and resilience on anxiety among older Australians. Aust. J, Psychol. 69, 263–272. 10.1111/ajpy.12152

[B35] Liao P. Chen S. Lin S. S. J. (2018). Latent profiles of stress and their relationships with depression and problematic Internet use among college freshmen. Scand. J. Psychol. 59, 621–630. 10.1111/sjop.12489 30252933

[B36] Löwe B. Decker O. Müller S. Brähler E. Schellberg D. Herzog W.. (2008). Validation and standardization of the Generalized Anxiety Disorder Screener (GAD-7) in the general population. Med. Care. 46, 266–274. 10.1097/MLR.0b013e318160d093 18388841

[B37] Lundt A. Jentschke E. (2019). Long-term changes of symptoms of anxiety, sepression, and fatigue in cancer patients 6 months after the end of Yoga therapy. Integr. Cancer Ther. 18, 1534735418822096. 10.1177/1534735418822096 30791735 PMC7240880

[B38] Luthar S. S. Cicchetti D. Becker B. (2000). The construct of resilience: a critical evaluation and guidelines for future work. Child Dev. 71, 543–562. 10.1111/1467-8624.00164 10953923 PMC1885202

[B39] Ma X. Wang Y. Hu H. Tao X. G. Zhang Y. Shi H. (2019). The impact of resilience on prenatal anxiety and depression among pregnant women in shanghai. J. Affective Disord. 250, 57–64. 10.1016/j.jad.2019.02.058 30831542

[B40] Martin R. A. Kazarian S. S. Breiter H. J. (1995). Perceived stress, life events, dysfunctional attitudes, and depression in adolescent psychiatric inpatients. J. Psychopathol. Behav. 17, 81–95. 10.1007/BF02229205

[B41] Monique C. Hibah O. Georges N. Ziyad M. (2010). Validation of the Arabic version of the Cohen perceived stress scale (PSS-10) among pregnant and postpartum women. BMC Psychiatry. 10:111. 10.1186/1471-244X-10-111 21159169 PMC3016315

[B42] Preis H. Mahaffey B. Heiselman C. Lobel M. (2020a). Pandemic-related pregnancy stress and anxiety among women pregnant during the COVID-19 pandemic. Am. J. Obstet. Gynecol. 100155. 10.1016/j.ajogmf.2020.100155 PMC7295479 32838261

[B43] Preis H. Mahaffey B. Heiselman C. Lobel M. (2020b). Vulnerability and resilience to pandemic-related stress among U.S. women pregnant at the start of the COVID-19 pandemic. Soc. Sci. Med. 266:113348. 10.1016/j.socscimed.2020.113348 32927382 PMC7474815

[B44] Romero-Gonzalez B. Puertas-Gonzalez J. A. Marino-Narvaez C. Peralta-Ramirez M. I. (2021). Confinement variables by COVID-19 predictors of anxious and depressive symptoms in pregnant women. Med. Clin. 156, 172–176. 10.1016/j.medcle.2020.10.010 33243419 PMC7832526

[B45] Rosenthal L. Earnshaw V. Lewis T. T. Reid A. E. Lewis J. B. Stasko E. C.. (2015). Changes in experiences with discrimination across pregnancy and postpartum: Age differences and consequences for mental health. Am. J. Public Health. 105, 686–693. 10.2105/AJPH.2014.301906 24922166 PMC4264991

[B46] Rutter M. (1985). Resilience in the face of adversity: protective factors and resistance to psychiatric disorder. Br. J. Psychiatry. 147:598–611. 10.1192/bjp.147.6.598 3830321

[B47] Saadati N. Afshari P. Boostani H. Beheshtinasab M. Abedi P. Maraghi E. (2021). Health anxiety and related factors among pregnant women during the COVID-19 pandemic: a cross-sectional study from Iran. BMC Psychiatry. 21:95. 10.1186/s12888-021-03092-7 33588794 PMC7883951

[B48] Sagone E. Caroli M. E. D. (2013). Relationships between resilience, self-efficacy, and thinking styles in Italian middle adolescents. Pro-Soc. Behav. Sci. 92, 838–845. 10.1016/j.sbspro.2013.08.763

[B49] Schwarzer R. Warner L. M. (2013). Perceived self-efficacy and its relationship to resilience, in Resilience in Children, Adolescents, and Adults: Translating Research Into Practice, eds Prince-Embury A. Saklofske D. H. (New York, NY: The Springer Series on Human Exceptionality, Springer), 139–150.

[B50] Selye H. (1950). Stress and the general adaptation syndrome. Br. Med. J. 1, 1383–1392. 10.1136/bmj.1.4667.1383 15426759 PMC2038162

[B51] Solomou I. Constantinidou F. (2020). Prevalence and predictors of anxiety and depression symptoms during the COVID-19 pandemic and compliance with precautionary measures: age and sex matter. Int. J. Environ. Res. Public Health. 17:4924. 10.3390/ijerph17144924 32650522 PMC7400373

[B52] Spitzer R. L. Kroenke K. Williams J. B. W. Löwe B. (2006). A brief measure for assessing generalized anxiety disorder. Arch. Intern. Med. 166:1092. 10.1001/archinte.166.10.1092 16717171

[B53] Stein A. Pearson R. M. Goodman S. H. Rapa E. Rahman A. McCallum M.. (2014). Effects of perinatal mental disorders on the fetus and child. Lancet 384, 1800–1819. 10.1016/S0140-6736(14)61277-0 25455250

[B54] Tam C. C. Benotsch E. G. Weinstein T. L. (2020). Resilience and psychiatric symptoms as mediators between perceived stress and non-medical use of prescription drugs among college students. Am. J. Drug Alcohol Ab. 46, 120–130. 10.1080/00952990.2019.1653315 31442086

[B55] Taylor J. M. (2015). Psychometric analysis of the Ten-Item Perceived Stress Scale. Psychol. Assess. 27, 90–101. 10.1037/a0038100 25346996

[B56] Tsourtos G. Ward P. R. Miller E. R. Hill K. Barton C. Wilson C. J.. (2019). Does resilience moderate the relationship between stress and smoking status? Subst. Use Misuse 54, 412–425. 10.1080/10826084.2018.1501066 30638106

[B57] Vollrath M. E. Sengpiel V. Landolt M. A. Jacobsson B. Latal B. (2016). Is maternal trait anxiety a risk factor for late preterm and early term deliveries? BMC Pregnancy Childbirth. 16:286. 10.1186/s12884-016-1070-1 27680098 PMC5041314

[B58] Waller M. A. (2001). Resilience in ecosystemic context: evolution of the concept. Am. J. Orthopsychiat. 71, 290–297. 10.1037/0002-9432.71.3.290 11495331

[B59] Wu Y. Zhang C. Liu H. Duan C. Li C. Fan J.. (2020). Perinatal depressive and anxiety symptoms of pregnant women during the coronavirus disease 2019 outbreak in China. Am. J. Obstetr. Gynecol. 223, 240.e1–240.e9. 10.1016/j.ajog.2020.05.009 32437665 PMC7211756

[B60] Yan H. Ding Y. Guo W. (2020). Mental health of pregnant and postpartum women during the coronavirus disease 2019 pandemic: a systematic review and meta-analysis. Front. Psychol. 11:617001. 10.3389/fpsyg.2020.617001 33324308 PMC7723850

[B61] Zemestani M. Fazeli Nikoo Z. (2020). Effectiveness of mindfulness-based cognitive therapy for comorbid depression and anxiety in pregnancy: a randomized controlled trial. Arch. Women Ment. Hlth. 23, 207–214. 10.1007/s00737-019-00962-8 30982086

[B62] Zimmerman M. A. Brenner A. B. (2010). Resilience in adolescence: overcoming neighborhood disadvantage, in Handbook of Adult Resilience, eds Reich J. W. Zautra A. J. Hall J. S. (New York, NY: The Guilford Press), 283–308.

[B63] Zimmerman M. A. Steinman K. J. Rowe K. J. (1998). Violence among urban African American adolescents: the protective effects of parental support, in Addressing Community Problems: Psychological Research and Interventions, eds Arriaga X. B. Oskamp S. (Newbury Park, CA: Sage Publications, Inc), 78–103.

[B64] Zubin J. Spring B. (1977). Vulnerability-a new view of schizophrenia. J. Abnorm. Psychol. 86, 103–126. 10.1037/0021-843X.86.2.103 858828

